# Heterogeneous Claudin-1 Expression in Human Liver

**DOI:** 10.1002/hep.25910

**Published:** 2013-02-05

**Authors:** Helen J Harris, Garrick K Wilson, Stefan G Hübscher, Jane A McKeating

**Affiliations:** Institute of Biomedical Research and NIHR Liver Biomedical Research Unit, University of BirminghamBirmingham, United Kingdom

## To the Editor:

**Fig. 1:**
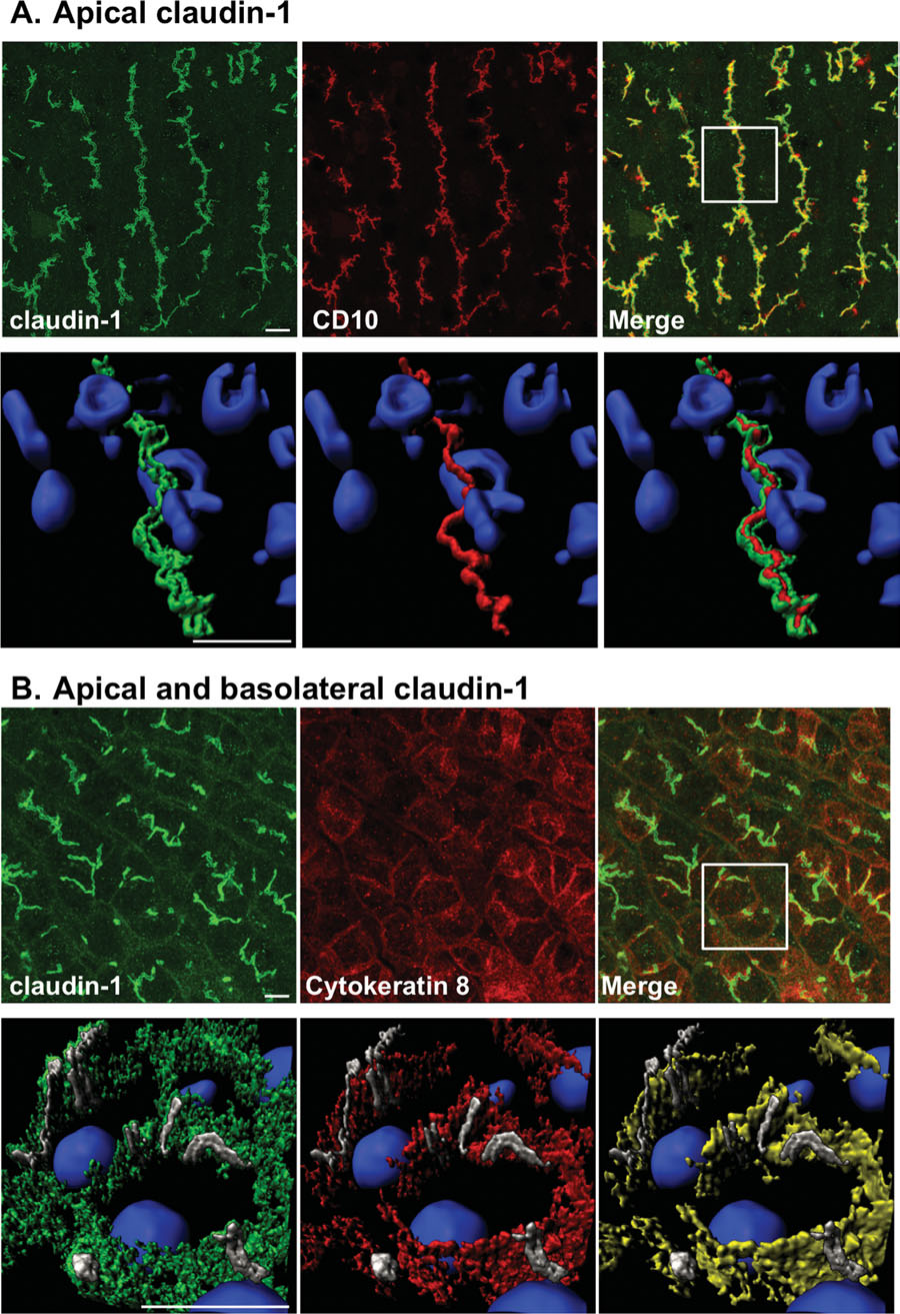
Claudin-1 localization in normal human liver. Representative images of claudin-1 costained with markers specific for apical (CD10) or basolateral (cytokeratin 8) membranes. Heterogeneous patterns of claudin-1 at apical and basolateral membranes were observed. Magnified volumetric images (white box) prepared using Imaris software demonstrate claudin-1 localization around CD10, in keeping with a pericanalicular tight junction distribution. Application of low and high fluorescent intensity thresholds discriminates apical tight junction-associated (silver) and basolateral (yellow) pools of claudin-1. Nuclei were stained with DAPI (blue) and the scale bar represents 10 μm. [Color figure can be viewed in the online issue, which is available at wileyonlinelibrary.com.]

We congratulate Mensa et al.^1^ on their report studying the expression of hepatitis C virus (HCV) receptors claudin-1 and occludin after liver transplantation and their influence on early viral kinetics. The authors provide a unique insight into the potential role receptor expression levels play in modulating early phase viral kinetics. They observed an association between HCV recurrence and hepatocellular claudin-1 and occludin expression levels expression during the first week post liver transplant. The authors confirm the results of previous reports showing increased claudin-1 expression in HCV-infected liver.^2^,^3^ However, Mensa et al. conclude that claudin-1 is solely located at the apical pole of hepatocytes, in contrast to reports by Reynolds et al. and Zadori et al.^2^,^3^ We agree that claudin-1 is predominantly expressed at the apical membrane of hepatocytes in normal liver; however, a minor pool of claudin-1 is observed at the basolateral membrane (Fig. 1). Basolateral expressed claudin-1 is more easily discerned when the liver section is co-stained with a marker for the hepatocellular membrane such as cytokeratin 8 (Fig. 1B), enabling one to observe heterogeneous patterns of localization across the liver parenchyma. The discrepancies between these studies are most likely explained by the imaging technique and analytical software employed. Spectral imaging of liver sections enables the accurate quantification of bound fluorescent antibody irrespective of signal intensity. However, volumetric imaging of claudin-1 at areas of high (apical) and low (basolateral) expression requires multiple threshold values (Fig. 1). In contrast, Mensa et al. quantified volumetric images of claudin-1 using a single threshold value, leading to a potential bias in their protein quantification and an underrepresentation of basolateral claudin-1. In conclusion, Mensa et al. have highlighted a role for viral receptor expression in defining HCV kinetics posttransplant, warranting further investigation to study the role of host pathways and inflammatory responses that regulate viral receptor hepatocellular expression.
